# Effects of Thermal Mass, Window Size, and Night-Time Ventilation on Peak Indoor Air Temperature in the Warm-Humid Climate of Ghana

**DOI:** 10.1155/2013/621095

**Published:** 2013-06-25

**Authors:** S. Amos-Abanyie, F. O. Akuffo, V. Kutin-Sanwu

**Affiliations:** ^1^College of Architecture and Planning, Kwame Nkrumah University of Science and Technology, Kumasi, Ghana; ^2^College of Engineering, Kwame Nkrumah University of Science and Technology, Kumasi, Ghana

## Abstract

Most office buildings in the warm-humid sub-Saharan countries experience high cooling load because of the predominant use of sandcrete blocks which are of low thermal mass in construction and extensive use of glazing. Relatively, low night-time temperatures are not harnessed in cooling buildings because office openings remain closed after work hours. An optimization was performed through a sensitivity analysis-based simulation, using the Energy Plus (E+) simulation software to assess the effects of thermal mass, window size, and night ventilation on peak indoor air temperature (PIAT). An experimental system was designed based on the features of the most promising simulation model, constructed and monitored, and the experimental data used to validate the simulation model. The results show that an optimization of thermal mass and window size coupled with activation of night-time ventilation provides a synergistic effect to obtain reduced peak indoor air temperature. An expression that predicts, indoor maximum temperature has been derived for models of various thermal masses.

## 1. Introduction 

**Figure 1:**
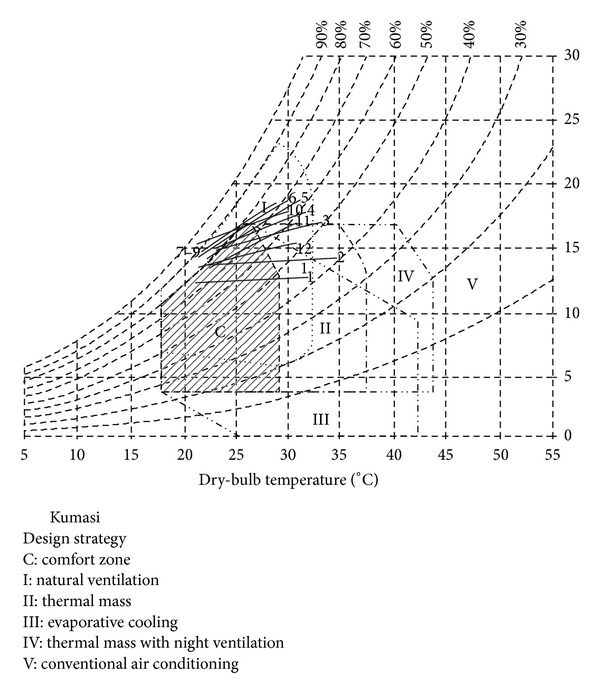
Building Bioclimatic Chart of Kumasi [1].

The predominant hot and humid tropical nature of the climate of Ghana makes climatic conditions predominantly fall outside the human comfort zone and thus require cooling for about 50 and 100% of the year for which air conditioning (AC) is used [1].  Figure 1 shows the Building Bioclimatic Chart for Kumasi, the second largest city in Ghana, showing the mean outdoor air temperature and relative humidity represented by the lines falling outside the comfort zone (shaded). The application of passive and low energy cooling techniques (PLECT) can reduce AC use to below 7% of the time of the year [1]; however, AC is adopted as a straightforward response to achieve thermal comfort without recourse to the potential of PLECT to reduce energy use in space cooling.

In recent years, there has been a movement towards universal building design approaches with extensive use of glazing that is poorly adapted to the local climatic conditions and also permits high penetration of solar radiation. Construction of building envelope in Ghana predominantly employs sandcrete blocks that are of low thermal mass. No empirical study has been carried out to assess its performance with that of other available materials. The relatively low night outdoor air temperature is not harnessed to cool the thermal mass of buildings. At night, there is reasonably very low air temperature; however, because the windows remained closed, as it is in normal practice in office buildings at night, the cool night air does not contribute to cooling the thermal mass at night. The cooling of the mass at night from the outside is hampered by the thermal resistance of the mass, and thus the inner layers are not cooled to provide a heat sink during the following day. The above leads to higher average peak indoor air temperatures with a corresponding higher cooling load.

There is lack of practical advice and empirical data on potential of PLECT to reduce peak air temperatures in figures readily appreciated by building designers and prospective builders. In many instances, the building design is not supported by a detailed analysis and evaluation of thermally relevant features and options related to orientation, envelope, glazing ratio, shading devices, and thermal mass. Thus, design decision making is not sufficiently informed by pertinent expertise pertaining to energy-efficient building design methods and technologies [2]. Selection of materials for a building in Ghana has often been based on affordability, aesthetics, appearance, durability, and in some cases availability, other than functional, thermal performance, and environmental considerations.

It has been projected that Ghana will need more than 7 times its 2007 electric power capacity by 2020 if it should succeed in developing its economy into a middle income one [3]. It is clear that Ghana will have to expand and diversify installed energy capacity. However, expansion is costly and takes time. In the meantime, any energy resources that can be saved through efficiency or conservation can be used for other purposes.

The aim of this paper is to investigate, in a systematic way, using both simulation and experimental approaches, the effects of thermal mass, window size, and night ventilation technique to reduce peak indoor air temperature in the mode of building design in Ghana to reduce cooling load in buildings.

## 2. Methodology

In investigations involving cooling potential of passive cooling techniques, energy simulation models of the building need to be validated with scale measured data from a building designed to run on the technique in question so that retrofit strategies can be targeted to solve the building's energy problems [4]. During the last two decades, several calibration methodologies have been developed [4–6]. Most of these methods employed site measurement and monitoring of the building's performance. However, while this approach is appropriate for retrofit projects with a limited budget [7], no building is designed in Ghana to run on night ventilation technique from which data can be collected for the validation.

**Figure 2:**
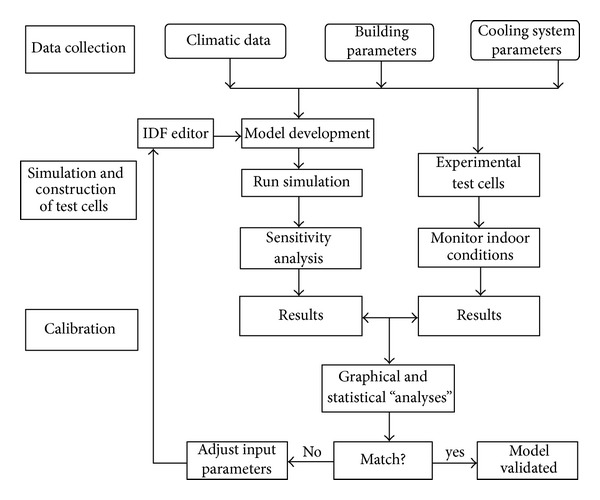
Flowchart of activities.

In the above context, the procedure employed in this study included the following tasks to achieve the aim of the research (Figure 2): (1) data collection of the climatic data and building parameters; (2) development of a simulation model and prediction of indoor air temperature; (3) design of experimental setup, construction, and monitoring of indoor air temperature; and (4) validation of the predicted (simulated) results with the experimental results employing graphical and statistical tools. Simulation was adopted to normalize confounding factors such as differences in obtained and actual weather file and construction mixtures [6]. Version 4 of Energy Plus Building Simulation Program for the analysis [8] was used.

### 2.1. Data Collection

The data collected included climatic data and building parameters. Simulation requires a detailed and comprehensive meteorological input data [5]. For this study, a weather file in a timestep of 6 was generated via Meteotest 2008 file [2]. The weather comprised outdoor dry-bulb temperature, relative humidity, wind speed, solar radiation, and ground temperature. The building parameters included the thermophysical properties of the basic building materials used for the model. Building parameters were obtained from the specifications and architectural drawings of the conventional mode of building design as specified in the Ghana Building Regulations. Building parameters helped to correctly dimension the model and specify zone data.

### 2.2. Simulation Model Development

A series of comparative simulations were performed using the weather file of Kumasi. The period for the simulation was from the month of November to March inclusively, a total of 212 days. This period represents the warmest months in Ghana [9]. A control model simulating the conventional mode of building design and construction in Ghana was first developed. The control models had dimensions of 2.40 m high, 1.2 m wide, and 2.4 m deep. The floor was made of 150 mm thick mass concrete, and the roof was made of 150 mm thick reinforced concrete slab. The wall of the control model was made of 150 mm thick solid sandcrete block which in this research represents the low mass material. The pilot survey of selected buildings in the region of study established the average window-to-floor ratio to be 54%. This was used as the base case of the control model. A total window-to-floor ratio of 1.62 m^2^ of glazing area was obtained with a floor area of 2.44 m^2^ of the control model. The control model had windows closed for 24 hours, with no night-time ventilation.

To assess the effect of thermal mass, three models were used, one with each of the three different thermal masses: solid sandcrete blocks (SSB), baked bricks (BB), and concrete (CONC) all with window-to-floor ratio of 54% as in Table 1. Nine models were used to assess the effect of window size. There were three models made of each of the solid sandcrete blocks (SSB), baked bricks (BB), and concrete (CONC). Among the three models of given thermal masses, each had 54% (window area of 1.62 m^2^), 27% (window area of 0.82 m^2^), and 0% window-to-floor ratios. The models used in assessing the effects of thermal mass and window size were not subjected to night-time ventilation. In assessing effect of night-time ventilation, the nine models used in assessing the effects of window size were subjected to a night-time ventilation rate of 10 ACH.  Table 2 shows the characteristics of models.

To assess the effect of varying night-time ventilation rate, the air change rates per hour were increased from 10 ACH to 20 ACH, then to 30 ACH. The test models were subjected to night-time ventilation from 19:00 hours (evening) till 7:00 hours (next morning). It is very common for night-time ventilation to be provided by natural ventilation. However, the site location of this research had irregular wind patterns and low wind speeds. This could not have provided effective natural ventilation rates for effective night-time ventilation. For this reason, mechanical ventilation was adopted with the use of an intake fan and an exhaust fan to provide night-time ventilation. During the daytime periods, the fans were provided with dampers to avoid internal heat gains by convection. Intake and exhaust ventilation fans were modelled for each model. 

### 2.3. Performance Evaluation of Simulation Models

The performance of the test models was evaluated using that of the control model as a baseline. Performance of the models was rated by evaluation of three variables: (1) the comparison of the maximum temperatures, (2) the temperature difference ratio (TDR), and (3) the percentage of overheated hours [10]. The temperature difference between a test model and the control model was indicative of the performance of the test model. According to Geros et al. [11], when the temperature reduction is high, the peak demand is delayed, and thus cooling load of a building is lower. The higher the drop in the peak indoor temperature of a test model, the better its performance.

The concept of TDR was proposed by Givoni and has been used with good results to compare passive cooling systems with different configurations [10]. TDR was calculated using the following expression:

(1) $$\begin{array}{c} \text{TDR}\;=\;\frac{\left(T_{\text{maxout}}-T_{\text{maxin}}\right)}{\left(T_{\text{maxout}}-T_{\text{minout}}\right)}, \end{array}$$

where TDR is temperature difference ratio; *T*_maxin_ is maximum temperature inside; *T*_maxout_ = maximum temperature outside; and *T*_minout_ is minimum temperature inside.

The numerator is the difference between the indoor maximum temperature and the outdoor maximum, and the denominator is the outdoor temperature swing. The higher the value of the TDR, the better the performance of a test model. A higher value indicates that there is a larger temperature difference between outdoors and indoors and there is more cooling. A negative TDR value indicates that the average maximum temperature inside the test cell is higher than that of outdoors. The relation between window-to-floor-ratio (WFR) and TDR was used to derive an expression to predict maximum indoor air temperature.

The number and percentage of overheated hours beyond the comfort band for each model were assessed. The comfort band proposed by Givoni [12] of between 18 and 29°C for tropical countries was used. Mean maximum temperature for a given day that was above 29°C were considered hot or uncomfortable. The number of hot and comfortable hours and their distribution permitted the examination of the overall pattern of temperature in the experimental models. A matrix made up of 24 rows that represent each hour of the day and a number of columns equal to the number of measured days in a series was prepared and used for the assessment of the performance of the models. Each of these cells indicated the average temperature for each hour of each day. The larger the difference in the number or percentage of overheated hours between a given test model and the control model the better the performance of the model. 

### 2.4. Experimental Setup

**Figure 3:**
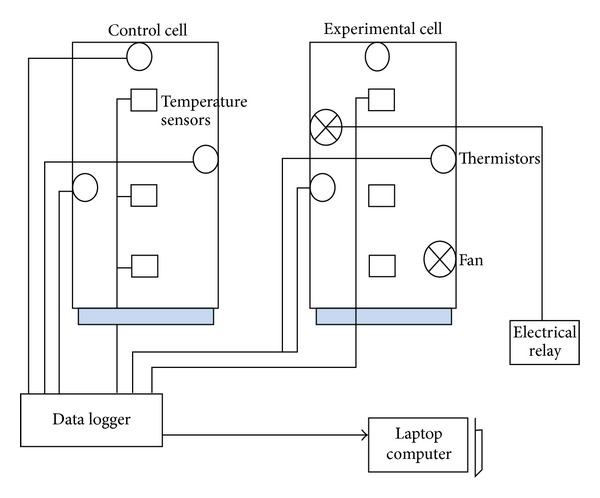
Schematic drawing of the experimental setup.

**Figure 4:**
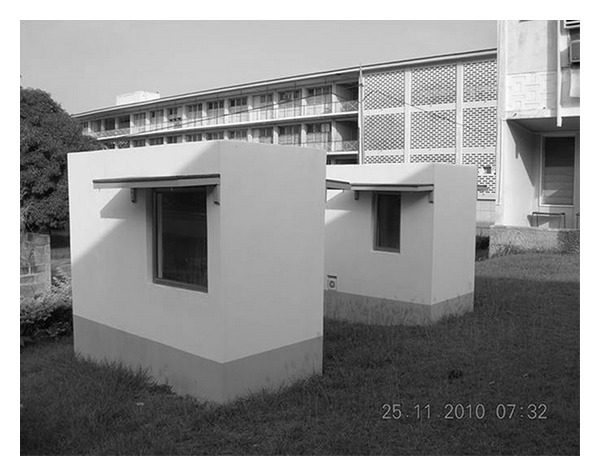
Test cells with installations.

An experimental setup was designed and constructed on the premises of the College of Architecture (CAP) at the Kwame Nkrumah University of Science and Technology (KNUST) in Kumasi, Ghana. The experimental system consisted of two cells (control and experimental cell); an active ventilation system consisting of two sets of 100 mm fan (in the south wall) and 100 mm outlet fans (in the north wall); HOBO H8 temperature data loggers to record air temperature; insulated external probe thermistor to monitor radiant temperature of internal surfaces. Figures 3 and 4, respectively, show the schematic drawing and the test cells with installations.

The test cells were carefully designed with the same specifications as the numerical models to achieve accurate experimental test results. The control and experimental cells had features of the base model and the most promising test model, respectively. The openings of both cells remained closed for 24 hours in a day.

The fans were operated to ventilate the experimental cell each night from 7 pm till 7 am (local time). The control cell was not ventilated at night. All the measured data were transferred through a USB to the laptop computer for the analysis. An area-weighted value for mean radiant temperature based on the five measured surfaces was calculated [13]:

(2) $$\begin{array}{c} T_{mr}=\frac{\sum_{1}^{4}T_{sn}A_{n}}{\sum_{1}^{4}A_{n}}, \end{array}$$

where *T*_*mr*_ is the mean radiant temperature; *T*_*sn*_ is the temperature of a surface; and *A*_*n*_ is the area of a surface.

### 2.5. Validation Procedure

Measured indoor air and radiant temperature from the test cells were used to calibrate the predicted (simulated) indoor air temperature and mean surface temperature of a two-week period for a satisfactory agreement. Statistical analysis involving the root mean square difference (*r* ^2^) and the corresponding coefficient of variance of root-mean-squared error (CV (RMSE)) value between simulated and measured air temperature and mean radiant temperature was computed and checked to fall within allowable tolerances. The coefficient of variance of the root-mean-squared error, CV (RMSE) (%), is essentially the root-mean-squared error divided by the measured mean of all the data, which is a convenient way of reporting a nondimensional result [14]. CV (RMSE) allows one to determine how well a model fits the data; the lower the CV (RMSE), the better the calibration. According to Kreider and Habel [15], the acceptable range considered appropriate for hourly CV (RMSE) of empirical models is 10 to 20%

(3) $$\begin{array}{c} \text{CV}\left(\text{MSE}\right)=\frac{{\left[{\left(\sum y_{\text{pred},\;i}-\sum y_{\text{data},\;i}\right)}^{2}/\left(n-p\right)\right]}^{1/2}}{{\overset{`}{y}}_{\text{data}}\times 100}, \end{array}$$

where *y*_pred, *i*_ is a predicted dependent variable value for the same set of independent variables; *y*_data, *i*_ is a data value of the dependent variable corresponding to a particular set of the independent variables; ${\overset{`}{y}}_{\text{data}}$ is the mean value of the dependent variable of the data set; *n* is the number of data points in the data set; and *p* is the total number of regression parameters in the model (arbitrarily assigned as 1 for all models).

## 3. Discussion of Simulation Results

Analysis of the simulated results shows that all the models, both control and test, maintained indoor maximum air temperatures above the outdoor air temperature. However, all the test models had improved conditions than the control model. The effects of thermal mass and window size were assessed with maximum temperature difference between test models and the control model, and the effects of night-time ventilation were assessed with maximum temperature difference, temperature difference ratio, and percentage of overheated hours.

### 3.1. Effect of Thermal Mass

From the analysis, increasing the thermal mass by changing to materials of higher densities led to a reduction in peak indoor air temperature. Concrete had the highest effect in reducing PIAT, followed by baked bricks and then solid sandcrete blocks.  Table 3 presents the peak indoor air temperature, maximum temperature differences with the control model, and peak delay hours of the various thermal masses tested. The control model made of the solid sandcrete blocks had a peak indoor air temperature of 35.6°C. The test models of baked bricks and concrete envelopes had an average peak indoor air temperature of 34.9°C and 32.6°C. This means that BB reduced peak indoor air temperature (PIAT) by 0.7°C and concrete had the highest effect with a drop of 3°C below that of the control model.

Increased thermal mass also led to an increase in the number of hours of delay of PIAT occurrence after peak outdoor air temperature. SSB, BB, and CONC had number of hours of delay of 2, 3, and 5 hours, respectively. The above indicates that walls made of concrete and baked bricks can maintain more steady indoor temperature with lower fluctuations and postpone the time of occurrence when peak indoor air temperature appears with a longer time lag than solid sandcrete blocks. Heat storage properties of concrete and baked bricks can postpone the time of occurrence when peak indoor air temperature appears with a longer time lag than solid sandcrete blocks.

In models of solid sandcrete block walls, indoor air temperature rises dramatically during the morning (early) hours and reaches its maximum in late afternoons. It then cools down rapidly in the evening (late) hours, with a wider fluctuation in diurnal indoor temperature pattern. The temperatures in the solid sandcrete block wall models closely followed that of the outdoor conditions and did not offer any significant thermal storage. This is because they are of low mass material. This observation is similar to observations made by [16] in an architectural science inquiry on four environmental test chambers with different thermal mass level types in Nairobi, Kenya. The models with baked bricks walls have mean indoor patterns that follow closely that of the solid sandcrete blocks but with relatively smaller fluctuations. 

The analysis revealed that higher thermal mass reduced indoor maxima and at the same time brought up indoor minima. However, the influence on maxima was larger than that on minima. In the test models with concrete walls, the moderating effect of thermal mass was noticeable. Because they are of a high mass material, the maximum indoor air temperatures are suppressed at daytime and rise slowly to achieve a relatively lower maximum in the late afternoons. The stored heat in concretes reradiates back into the space at night and causes much higher minimum temperature during night hours. The observed trend is characteristic of high mass materials, and similar observations were made in a study by [17] to investigate the effects of colour and building thermal mass in the hot humid climate of Hong Kong. Indoor temperature fluctuations associated with baked bricks and concrete are kept to a minimum.

### 3.2. Effect of Window Size

Reduced window-to-floor ratio for all the envelope materials tested leads to reduction in peak indoor air temperature for all the thermal masses (Table 4). Concrete wall envelope with no window had the best performance with the lowest PIAT. However, windows are an important source of natural lighting and views from nature that should not be eliminated in buildings.

The reductions were, however, not remarkable with none being up to 0.9°C drop in PAIT. This can be attributed to the effect of shading devices on the test models which eliminates solar and conductive heat gains through the windows. The effective shading led to marginal effect of the varying window sizes. A study by La Roche and Milne [10] to quantify the effects of modifying the amount of thermal mass and the window area on indoor comfort using smart controllers revealed that an increase rather in the size of unprotected window could have a more significant the performance.

### 3.3. Effect of Night-Time Ventilation

An analysis of maximum temperature difference between night-time ventilated models and the control model revealed that the largest difference of 4.3°C was obtained in the concrete model (model 9) that has no window and heavy weight mass with night ventilation (Table 5). The test models of series 3, 6, 7, and 8 also have appreciable differences between their average maximum and that of the control model that indicates that features in these test models have a significant effect on their performance. These features are the reduction of the window size, more thermal mass, or night ventilation. But it is the combination of these features that achieves the improved performance.

The impact of night ventilation was assessed for various air flow rates considering 10, 20, and 30 ACH. Increase in the air change rate did insignificantly reduce average peak indoor air temperature. For instance, for model 9, increase in air rate per hour from 10 to 20 ACH and from 20 to 30 ACH leads to reductions of 0.067°C and 0.044°C, respectively. Models 3, 6, 7, and 8 also had significant features with mean reductions of 0.046°C and 0.016°C with increased air rate per hour from 10 to 20 ACH and from 20 to 30 ACH. However, night ventilation of BB and CONC exposed them to cool outdoor air to offset, in part, the reradiated heat. This provides a relatively lower minimum indoor air temperature at night. The attained low indoor air temperature and cooled mass provided a heat sink that suppressed the attainable maximum indoor temperature during the daytime.

Unlike a study by [18] in the hot-humid climates at the Passive Cooling Laboratory (PCL) in the Florida Solar Energy Centre where increased air exchange rate could lower the average indoor air temperature, the difference in temperature as a result of night-time ventilation between the low mass and the high mass walls for this study was negligible. This could be explained by the relatively lower diurnal temperature range of Kumasi which does not effectively cool the building mass.

**Figure 5:**
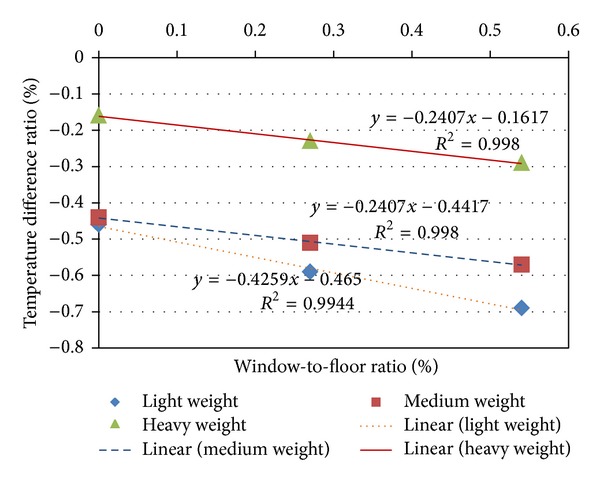
TDR as a function of the window-to-floor ratio.

Temperature difference ratio (TDR) was calculated and averaged for all the days for each model (Table 5). The best TDR amongst the test models is model 9, which performs far better, over four times, than the control model. Amongst all the thermal masses, as the north and south window-to-floor ratio increases, the TDR decreases. This implies that the large south- and-north facing windows reduce the performance of the test models. The relation between TDR and window-to-floor ratio (Figure 5) for the different thermal masses provided basis for the development of the predictive expression that can be used to predict TDR as a function of the floor to window ratio is derived (4). When TDR of a building is calculated for a given material, it is possible to predict the indoor maximum temperature using (1) and solving for *T*_maxin_, where outdoor maximum and minimum temperatures, or daily temperature swing, must be known.

Since no trend line strikes the positive window-to-floor area axis, it means that for all masses, conditions inside the models would be higher (or worse) and above the average outdoor temperatures even when there are no windows. In La Roche and Milne [10] study, when the south window-to-floor ratio was of a minimum of 11% and above, the indoor air conditions in the control cell, with a fixed infiltration rate, were higher (worse) than that of the average outside air temperature. While the predictive expression developed for indoor maximum temperatures in this research is similar to that by La Roche [19] in Lyle Centre in the Cal Poly Pomona, differences exist in the gradient and the constants. The differences between the expressions could be attributed to differences in thermal properties of the materials and climatic conditions associated with this study and that of [10].

 Light-weight mass:
  (4) TDR=−0.4259∗WFR−0.465 [R2=0.99]
 Medium-weight mass:
  (5) TDR=−0.2407∗WFR−0.4417 [R2=0.98]
 Heavy-weight mass:
  (6) TDR=−0.2407∗WFR−0.1617 [R2=0.99].

In all the above, TDR is a temperature difference ratio; WFR is window-to-floor ratio (north and south window).

The variation in the expressions for this study suggests that it is applicable to buildings of envelopes of the respective thermal masses of 150 mm thick mass walls, with shaded north and south windows only, and 150 mm mass concrete floor construction, and night ventilated at 10 ACH in the warm-humid climates similar to that of the Ashanti Region of Ghana.

For all the expressions, as window-to-floor ratio increased, the temperature difference ratio decreased. 

The number of overheated hours of each model for the simulation period is given in Table 5 and is compared with the number for the control model. With the base temperature of 29°C, the achieved reduction of the overheating hours for sandcrete blocks varies between 36% and 42% for air flow rate of 10 ACH. For baked bricks, the corresponding decrease varies between 37% and 39%, while for concrete, the reduction is between 35% and 39%.

## 4. Validation (Calibration) of Simulation Results

**Figure 6:**
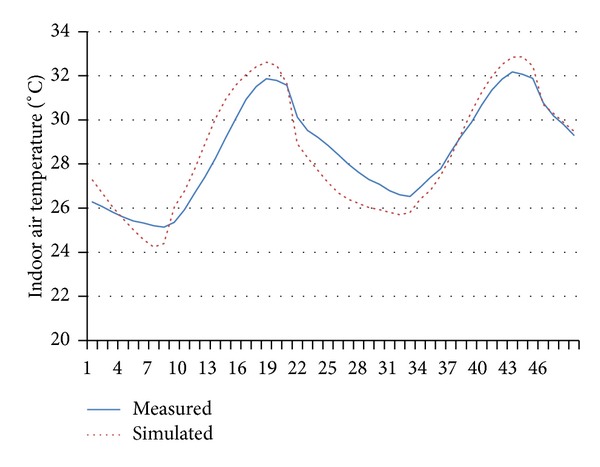
Measured versus simulated indoor air temperatures.

**Figure 7:**
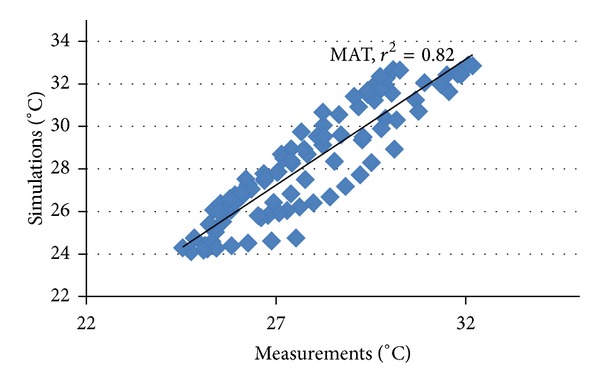
Measured versus simulated indoor air temperatures.

Predicted values of the simulation models compared well with the measured values. Figures 6 and 7 illustrate segments of the match and relationship (in terms of regression lines) measured versus simulated indoor air temperature, respectively. The respective correlation coefficient values are summarized in Table 6. In terms of statistic analyses, root-mean-square difference (*r* ^2^) was 0.82 and 0.83 for indoor air temperature and mean radiant temperature, respectively, and coefficient of variation for the root-mean-squared error (CV (RMSE)) was 14.75% and 16.80% for indoor air temperature and mean radiant temperature, respectively, and is considered statistically inappropriate falling within the range suggested by [15]. The good match of predicted results with the measured data suggests that thermophysical properties of the materials used are consistent with that specified in the simulation program.

## 5. Conclusion

Employing both sensitivity-based simulation and experimental approaches, the paper has presented the results of the effects of thermal mass, window size, and night-time ventilation on peak indoor air temperature in the warm-humid climate conditions with Ghana as a case.

From the analysis, increasing the thermal mass by changing to materials of higher densities led to a reduction in peak indoor air temperature. Concrete had the highest effect in reducing PIAT and maintained a drop of 3°C below that of the solid sandcrete blocks, with baked bricks having a drop of 0.7°C. Increased thermal mass also led to an increase in the number of hours of delay of PIAT occurrence after of peak outdoor air temperature. Solid sandcrete blocks, baked bricks, and concrete had number of hours of delay of 2, 3, and 5 hours, respectively. The above indicates that walls made of concrete and baked bricks have heat storage properties that can maintain more steady indoor temperature with lower fluctuations and postpone the time of occurrence when peak indoor air temperature appears with a longer time lag than solid sandcrete blocks. 

A decrease in window size leads to reduction in peak indoor air temperature for all the envelope materials (thermal masses) tested. The reductions were, however, not remarkable with none being up to 0.9°C drop in PIAT. This can be attributed to the effect of shading devices on the test models which eliminates solar and conductive heat gains through the windows. The effective shading led to marginal effect of the varying window sizes. Concrete wall envelope with no window had the best performance with the lowest PIAT. However, windows are an important source of natural lighting and views from nature that should not be eliminated in buildings. Since no trend line strikes the positive window-to-floor area axis of the TDR-WFR correlation, it means that for all masses, conditions inside the models would be higher (or worse) and above the average outdoor temperatures even when there are no windows.

The analysis of indoor air temperature has shown that at an air change rate per hour of 10 ACH for various window-to-floor ratios, the expected reduction of the overheated hours was between 36% and 42%, 37% and 39%, and 37% and 39% for sandcrete blocks, baked bricks, and concrete, respectively. Increase in the air change rate per hour to 20 ACH and then to 30 ACH did not significantly reduce peak indoor air temperature. 

The good match observed on validation of the of predicted results with the measured data confirms that the thermophysical properties of basic materials used for construction in Ghana are consistent with that used in international standard building simulation programs. 

It can be concluded that the combined effects of thermal mass, window size, and night-time ventilation can synergistically reduce peak indoor air temperature in the warm-humid climates and improve thermal comfort. With the current leanings towards environmental sustainability of building design, the study is valuable towards energy-efficient building design in Ghana. These findings could guide building designers, prospective builders, and facility managers in the selection of building envelope material that will improve thermal performance and achieve thermal comfort. Future research is required to investigate the impact of other building envelop materials in Ghana on peak indoor temperature in buildings. Theoretically, the findings from this paper contribute further to the study of knowledge by providing sufficient evidence to support earlier studies that night-time ventilation is not effective in some warm-humid environments with narrow diurnal temperature range.

## Figures and Tables

**Table 1:** Treatments made to the control model.

Thermal mass	SSB			BB			CONC		
Model no.	**1 **	**2 **	**3 **	**4 **	**5 **	**6 **	**7 **	**8 **	**9 **
W-F-R	54%	27%	0	54%	27%	0	54%	27%	0
Effect of mass	*√*			*√*			*√*		
Effect of window size	*√*	*√*	*√*	*√*	*√*	*√*	*√*	*√*	*√*
Effect of NV	*√*	*√*	*√*	*√*	*√*	*√*	*√*	*√*	*√*

**Table 2:** Description of models.

Test model	Description
Model 1	Light-weight mass with 0.54% window-to-floor ratio
Model 2	Light-weight mass with 0.27% window-to-floor ratio
Model 3	Light-weight mass with no window
Model 4	Light-weight mass with 0.54% window-to-floor ratio
Model 5	Light-weight mass with 0.27% window-to-floor ratio
Model 6	Light-weight mass with no window
Model 7	Heavy-weight mass with 0.54% window-to-floor ratio
Model 8	Heavy-weight mass with 0.27% window-to-floor ratio
Model 9	Heavy-weight mass with no window

**Table 3:** Outdoor and indoor air temperatures of model 9 and the control model.

	PIAT^a^	DBC^b^	PDH^c^
MAX_out _	30.1	—	

SSB	35.6	—	2
BB	34.9	0.7	3
CONC	32.6	3.0	5

^a^Max. indoor air temperature.

^b^Difference below control Model.

^c^Peak delay hours.

**Table 4:** Indoor air temperature models with varied window patterns.

Window-to-floor ratio	PIAT			DBC		
	SSB	BB	CONC	SSB	BB	CONC
54%	35.58	34.93	32.56	0.00	0.66	3.03
27%	34.77	34.16	32.06	0.81	1.42	3.52
No window	33.78	33.63	31.52	1.80	1.95	4.06

DAO: difference above outdoor maximum temperature.

DBC: difference below control model.

**Table 5:** Maximum temperature differences, temperature difference ratios, and percentage of overheated hours.

Models	Control	SSB			BB			CONC		
		1	2	3	4	5	6	7	8	9
MEAN MAX. (Nvent—10 ACH)	35.58	35.56	34.74	33.73	34.61	344.13	33.59	32.39	31.87	31.31
10 ACH	—	0.026	0.849	1.849	0.975	1.453	1.990	3.191	3.710	4.278
20 ACH	—	0.033	0.857	1.860	0.983	1.462	2.001	3.244	3.770	4.345
30 ACH	—	0.036	0.861	1.865	0.987	1.467	2.007	3.270	3.799	4.379
TDR at 10 ACH	—	−0.69	−0.59	−0.46	−0.57	−0.51	−0.44	−0.29	−0.23	−0.16
Overheated hours	48.2	41.1	38.7	36.9	39.3	38.3	37.5	38.7	37.5	35.7

**Table 6:** Correlation coefficient values.

	*r* ^2^	CV (RMSE)
Indoor air temperature	0.82	14.75%
Mean radiant temperature	0.83	16.80%
